# Optimizing immunofluorescence with high-dynamic-range imaging to enhance PD-L1 expression evaluation for 3D pathology assessment from NSCLC tumor tissue

**DOI:** 10.1038/s41598-024-65187-x

**Published:** 2024-07-02

**Authors:** Hsien-Neng Huang, Chun-Wei Kuo, Yu-Ling Hung, Chia-Hung Yang, Yu-Han Hsieh, Yu-Chieh Lin, Margaret Dah-Tsyr Chang, Yen-Yin Lin, Jen-Chung Ko

**Affiliations:** 1 Department of Pathology, National Taiwan University Hospital Hsin-Chu Branch, Hsinchu, Taiwan; 2 Department and Graduate Institute of Pathology, College of Medicine, National Taiwan University, Taipei, Taiwan; 3 JelloX Biotech Inc., Hsinchu, Taiwan; 4 Department of Internal Medicine, National Taiwan University HospitalHsin-Chu Branch, No. 25, Ln. 442, Sec. 1, Jingguo Rd., North Dist., Hsinchu City, 300 Taiwan, ROC

**Keywords:** Non-small cell lung cancer, Programmed death ligand 1, Immunofluorescence, Three-dimensional pathology, High dynamic range, Biotechnology, Cancer, Computational biology and bioinformatics, Molecular biology, Biomarkers, Medical research, Molecular medicine, Materials science, Optics and photonics

## Abstract

Assessing programmed death ligand 1 (PD-L1) expression through immunohistochemistry (IHC) is the golden standard in predicting immunotherapy response of non-small cell lung cancer (NSCLC). However, observation of heterogeneous PD-L1 distribution in tumor space is a challenge using IHC only. Meanwhile, immunofluorescence (IF) could support both planar and three-dimensional (3D) histological analyses by combining tissue optical clearing with confocal microscopy. We optimized clinical tissue preparation for the IF assay focusing on staining, imaging, and post-processing to achieve quality identical to traditional IHC assay. To overcome limited dynamic range of the fluorescence microscope’s detection system, we incorporated a high dynamic range (HDR) algorithm to restore the post imaging IF expression pattern and further 3D IF images. Following HDR processing, a noticeable improvement in the accuracy of diagnosis (85.7%) was achieved using IF images by pathologists. Moreover, 3D IF images revealed a 25% change in tumor proportion score for PD-L1 expression at various depths within tumors. We have established an optimal and reproducible process for PD-L1 IF images in NSCLC, yielding high quality data comparable to traditional IHC assays. The ability to discern accurate spatial PD-L1 distribution through 3D pathology analysis could provide more precise evaluation and prediction for immunotherapy targeting advanced NSCLC.

## Introduction

Hematoxylin and eosin (H&E) staining, couple with immunohistochemistry (IHC) assays, have served as standard pathological assays since the mid-twentieth century^1,2^. Chromogenic IHC, which was predominantly applied in surgical pathology samples, facilitates visual quantitation and distribution of target biomarkers through the antibodies interacting with a peroxidase-activated chromogenic substrate, resulting in substrate precipitation^3^. As a standard for diagnosing the emerging biomarkers as programmed death ligand 1 (PD-L1), chromogenic IHC plays a crucial role in guiding immune-checkpoint therapy treatment selection^4,5^. Clinical biomarker guidelines currently rely on visible light dyes and pattern identification, and on experienced pathologists quantified for reading IHC slides under a bright field microscope^6^.

The immunofluorescence (IF) technique has gained popularity in scientific research and clinical laboratories for labeling specific biomarkers using fluorophores, owing to its high sensitivity in dark-field images^7,8^. Conjugated polymers applied to antibodies allow for the observation of biomarker localization and distribution in biological tissues using fluorescent microscopy^9,10^. Recently, tyramide signal amplification (TSA) technology has been developed to enhance the sensitivity of weak biomarker by detecting antigens with tyramide-conjugated fluorophores, similar to chromogenic IHC’s immunoperoxidase staining mechanism^11^. The specific excitation/emission spectra of each fluorophore enable multiplex staining, allowing for the simultaneous observation of multiple biomarkers^12,13^. Nevertheless, IF staining for protein expression assessment remains a potential technology for practical pathological diagnosis in digital pathology.

More recently, fluorescence microscopy has become the main tool for fluorescent signal imaging and pattern visualization in advanced biomedical applications^14^. It allows for the simultaneous detection of multiple signals assigned to specific tissue structures, proteins, or nucleic acids^15,16^. Digital fluorescence signals enable data analysis or processing using computers rather than traditional light microscopy^17,18^. The combination of tissue-clearing methods with IF staining and fluorescence confocal microscopy has made 3D histological imaging feasible, revealing the tumor microenvironment’s protein distribution at different tissue depths^19,20^. Though fluorescence microscopy boasts superior sensitivity compared to conventional microscopy, however, the challenges were cost performance ratios and complex processing procedures, which hinder its integration into clinical practice. Despite efforts to enforce excitation and emission spectra, the wide fluorescence intensity range can compromise imaging quality^11^. The demand of fluorescence images in advanced biological research is peremptorily increasing; therefore, several literatures focus on overcoming dynamic-range limitation in the fluorescence images with physical or digital improvement^21–24^. Additionally, manual interpretation and correlation between dark-field (IF) and bright-field (IHC) images pose challenges due to nonlinear human eye responses. Facing these challenges, we require advanced detector at an affordable expense for routine clinical practice. Standardized processes for staining, imaging, and post-processing of digital fluorescent images are crucial for the routine clinical pathologic analysis using fluorescence techniques.

We established a refined approach for staining, imaging, and post-processing PD-L1 IF digital images in NSCLC to achieve diagnostic quality akin to traditional IHC patterns. A comparative verification of IF and traditional IHC was conducted to standardize IF image interpretation. Finally, we extended this approach to 3D digital IF images with optical clearance to analyze PD-L1 expression variation at various tumor tissue depths which was not achieved by IHC tests.

## Materials and methods

### Specimens

All procedures were approved by the ethical committee of National Taiwan University Hospital Hsin-Chu Branch (Institutional Review Board 111-079-F). Sequential 4-μm-thick formalin-fixed, paraffin-embedded (FFPE) sections from 30 cases of NSCLC were prepared for conventional histological and IF assays.

### Chromogenic IHC and IF

IHC was performed using an automated staining system (VENTANA BenchMark XT; Roche) with antibodies against PD-L1 (clone SP263) (Ventana Medical Systems). IF assays were performed manually, and antigen retrieval (heat-induced epitope retrieval) was performed using Cell Conditioning Solution 1 (Ventana Medical Systems, 950-124). Slides were incubated with anti-PD-L1 antibodies for 1 h at room temperature. For IF assay using a fluorophore-conjugated secondary antibody (2′Ab), the slides were incubated for 1 h with Alexa Fluor 568–labeled polyclonal anti-rabbit antibody (dilution 1:500; Thermo Fisher Scientific, A11011). After washing, the sections were covered with 1X TrueBlack solution (Biotium, 23007) for 1.5 min and washed with phosphate-buffered saline. For IF assay using the TSA system, the slides were incubated with poly-horseradish peroxidase (HRP)-conjugated goat anti-rabbit IgG antibody (Thermo Fisher Scientific, B40923) for 1 h after primary antibody staining. The slides were incubated with Alexa Fluor 555 tyramide (dilution 1:100, Thermo Fisher Scientific, B40923) for 10 min, and counterstained with 4′,6-diamidino-2-phenylindole (DAPI; Sigma-Aldrich, D9542). Human placental and tonsil tissues were used as positive controls for PD-L1 expression (Supplementary Fig. S1).

### 3D IF staining

3D IF staining was performed with 100-μm-thick FFPE tissue sections. The sections were deparaffinized by immersion in Hemo-De (Scientific Safety Solvents, HD-150), followed by use of a downgraded series of ethanol solutions. After treated with 2% Triton (Sigma-Aldrich, V900502), antigen unmasking was performed using Uni-trieve (Innovex Biosciences Inc., NB325), followed by incubation with anti-PD-L1 (clone SP263) antibodies at 4 °C for 48 h. The sections were then incubated with poly-HRP-conjugated goat anti-rabbit IgG, followed by treatment with Alexa Fluor 555 tyramide, and counterstained with DiD (20 μg/mL; Thermo Fisher Scientific, D307) and SYTO-16 (5 μM; Thermo Fisher Scientific, S7578) to target lipid and nuclei, respectively. Finally, the sections were immersed in the clearing reagent (JelloX Biotech Inc., Hsinchu, Taiwan, JXClear DX) at 25 °C overnight followed by sealing in clearing reagent before image acquisition^25^.

### Image collection

A VENTANA DP 200 slide scanner (Roche Diagnostics) was used to obtain images of IHC slides. IF slides were scanned using SLIDEVIEW VS200 (Olympus) with a digital 12-bit camera in fluorescent observation mode. The slides were excited using an LaserLED Hybrid source (X-Cite TURBO, Excelitas Technologies) at wavelengths of 385 and 575 nm for DAPI and Alexa Fluor fluorescent dyes excitation, respectively. For DAPI excitation, the light source intensity and exposure time were fixed at 50% and 5 ms. The light source intensity and exposure time was fixed at 100% and 360 ms for Alexa Fluor 568 excitation. For TSA-stained slides, the light source intensity was fixed at 50% for Alexa Fluor 555 excitation, and the exposure time was arranged in order at 6.5, 25, and 55 ms, as in the original image for high dynamic range (HDR) algorithm. Whole slide image exporting was performed using Imaris 9.8 software (Bitplane).

3D histopathological imaging was performed with an FV3000 confocal microscope (Olympus) using a 20 × air objective lens. SYTO-16, DiD, and Alexa Fluor 555 were excited by 488 nm, 640 nm, and 561 nm lasers, respectively. The region of interest (ROI) in each sample was selected and acquired at a lateral resolution of 0.621 μm with a z-axis at an interval of 1-μm. The IHC images from the same tissue block of each were taken as a reference to the modified imaging condition of 3D IF images during image collection. Detailed conditions are shown in Supplementary Table S1. FV31S-DT software (Olympus) and Imaris 9.8 software was used for image stitching, image exporting, normalization, and 3D reconstruction.

### HDR algorithm

This study adapted the conventional HDR algorithm that was first proposed by Debevec and Malik^26^, where the modified version was specially designed for PD-L1 channels in IF microscopy applications. Four major specializations were included. First, prior to merging images of various exposures, the images were preprocessed by erosion and Gaussian blurring. The HDR algorithm consumed the original images along with their pre-processed counterparts, which were regarded as virtually created images with the same exposures. Second, when reconstructing the irradiance response curve during HDR, only pixels that were near the nuclei with significant PD-L1 expression were sampled for the computation. Third, the merged image was linearly scaled between the minimum and maximum PD-L1 expression over the input images. Fourth, after HDR processing, the merged image was post-processed by (a) luminance adjustment via a variant of gamma correction, (b) contrast-limited adaptive histogram equalization^27^, and (c) contrast enhancement using black-hat and white-hat transformations. More details are described in Supplementary Methods.

### Computer-vision approach and analysis

A computer-vision analysis was performed to estimate and compare the area proportion of PD-L1 expression in the annotated tumor regions of the IHC images, original IF images, and HDR-processed IF images. Fifteen specimens, consisting of five cases randomly chosen from each of the three tumor proportion score (TPS) categories (< 1%, 1–49%, or ≥ 50%), were included in the analysis. The tumor regions were manually annotated on both IHC and IF slides (Supplementary Fig. S2) by an experienced biology scientist. The area of PD-L1 expression in the annotated tumor regions of IHC images was estimated using the Macenko color deconvolution algorithm (details are shown in Supplementary Methods)^28^. The area proportion of PD-L1 expression was calculated from the number of pixels with values above the respective threshold of the image, along with the micrometer-per-pixel parameter, which was 0.465 and 0.334 for IHC and IF images, respectively. Despite no exact coincidence with the definition of PD-L1 TPS, we addressed that this expression area proportion could be a fair approximation.

### Interpretation of PD-L1 TPS

PD-L1 TPS of 30 cases was determined by two pathologists (HNH and CWK) according to the guidelines^6^. Scoring of IHC images, original IF images, and HDR images was performed in separately diagnosed rounds, followed by 2 weeks of wash-out period with random order between each round. The IHC images and exported IF images were viewed by two pathologists using Ventana Image Viewer v3.2 (Ventana) and a computer software MetaLite, developed by JelloX Biotech Inc., respectively. These cases were classified into three categories (TPS < 1%, 1–49%, or ≥ 50%). Two cases were excluded from the analysis because the number of tumor cells in the sections was < 100. Two pathologists were blinded to the scoring results. All scores are listed in Supplementary Table S2. Human tonsil tissues with IHC/IF and slides without primary antibodies were used as positive and negative controls, respectively.

The 3D fluorescent-stained ROI images with HDR processing were reviewed by pathologist HNH using MetaLite and determined the PD-L1 TPS of each image of the tissue.

### Statistical analysis

During computer-vision analysis for original IF images and HDR images, we performed one-sided Student’s t-tests on the absolute error of their PD-L1 expression area proportion to the IHC images. Weighted kappa was used to determine the concordance of TPS between IHC and IF images, and also assess interobserver reliability in classifying IHC and IF images according to TPS categorization. The reliability was interpreted as: poor (< 0), slight (0.01–0.20), fair (0.21–0.40), moderate (0.41–0.60), substantial (0.61–0.80), and almost perfect (0.81–1.00)^29^.

### Ethics declarations

All procedures were approved by the ethical committee of National Taiwan University Hospital Hsin-Chu Branch (Institutional Review Board 111-079-F) and was performed in accordance with the Helsinki Declaration. The research involved no more than minimal risk to subjects, and IRB approved a request to waive of all of the required elements of informed consent.

## Results

### Comparative analysis of PD-L1 expression patterns in IHC and IF images

**Figure 1:**
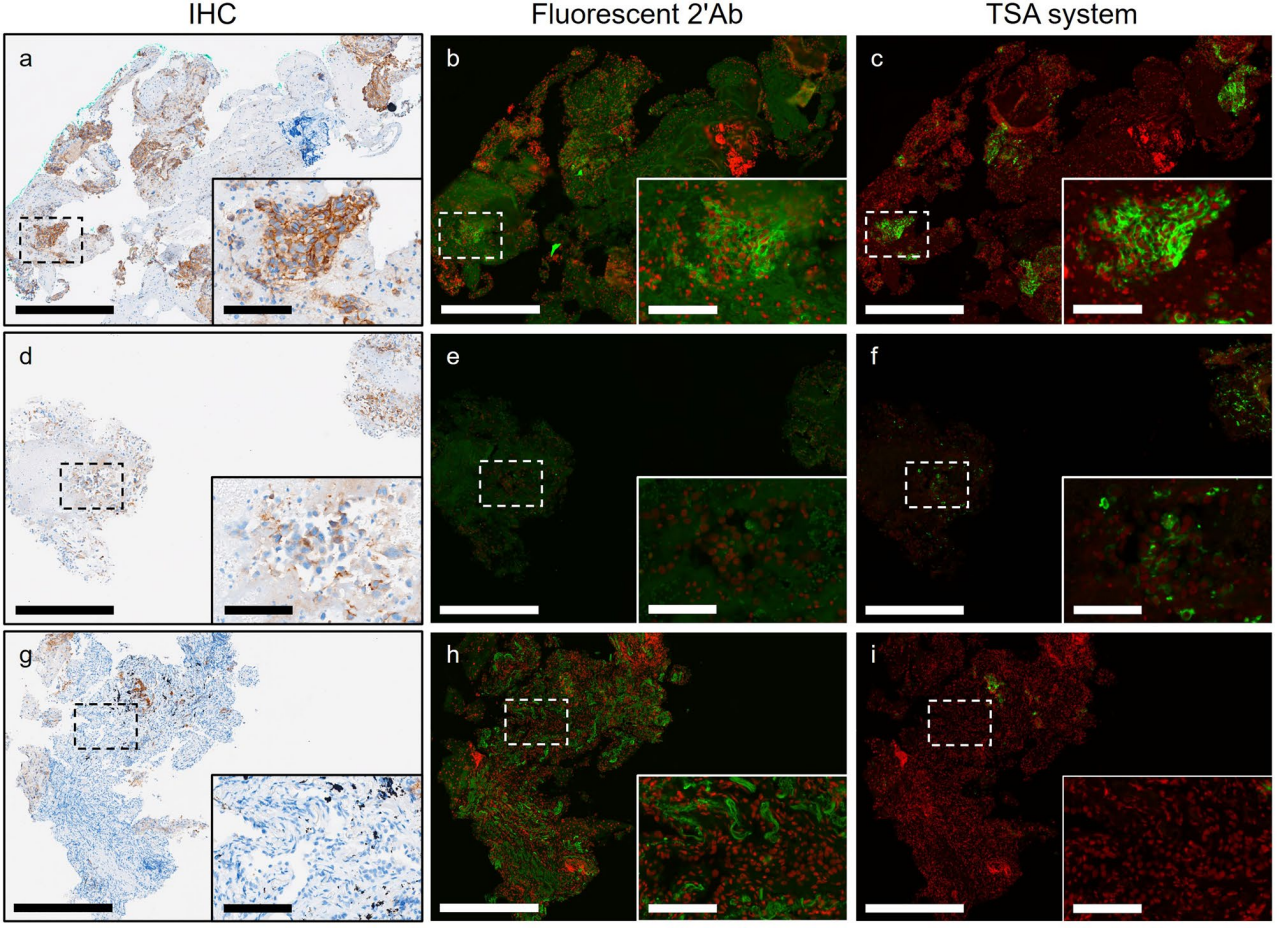
Expression patterns of PD-L1 in IHC and IF images of NSCLC sections. (**a**–**c**) Representative images from IHC, fluorescent 2′Ab-stained, and TSA-stained slides of tissue regions of NSCLC specimens with strong PD-L1 expression. Zoom-in detailed view of dashed box area shows in lower right corner of images. (**d**–**f**) Representative images from IHC, fluorescent 2′Ab-stained, and TSA-stained slides of tissue regions of NSCLC specimens with weak PD-L1 expression. (**g**–**i**) Representative images of NSCLC specimens from antigen-negative tissue regions from IHC, fluorescent 2′Ab-stained, and TSA-stained slides. Significant background signals showed in regions of NSCLC tissue in slides stained with fluorescent 2'Ab. *Green* PD-L1. *Red* nuclei. Scale bar: 500 μm; detailed view: 100 μm.

In previous IF-related studies, the TSA system demonstrated superior sensitivity for signal detection compared to indirect IF assay using fluorophore-conjugated antibody^30,31^, especially beneficial for targets with low antigen abundance^32–34^. To identify a method yielding quality comparable to clinical IHC assay, we compared the fluorescent 2′Ab method with the TSA system. As shown in Fig. 1a–c, both methods presented a complete membranous PD-L1 pattern in regions comparable to IHC with intense chromogen staining. In instances of weak PD-L1 expression detected in IHC, the TSA method preserved most of the PD-L1 pattern compared to the fluorescent 2′Ab method (Fig. 1d–f). Moreover, the fluorescent 2′Ab method exhibited noticeable background signals in normal tissue regions, such as blood cells and stroma (Fig. 1g–i). Although faint PD-L1 signals in IHC were slightly blurred in the TSA method, we selected the TSA method for subsequent studies to achieve practical pathological diagnosis quality.

### Evaluation of exposure time for standard IF imaging process

**Figure 2:**
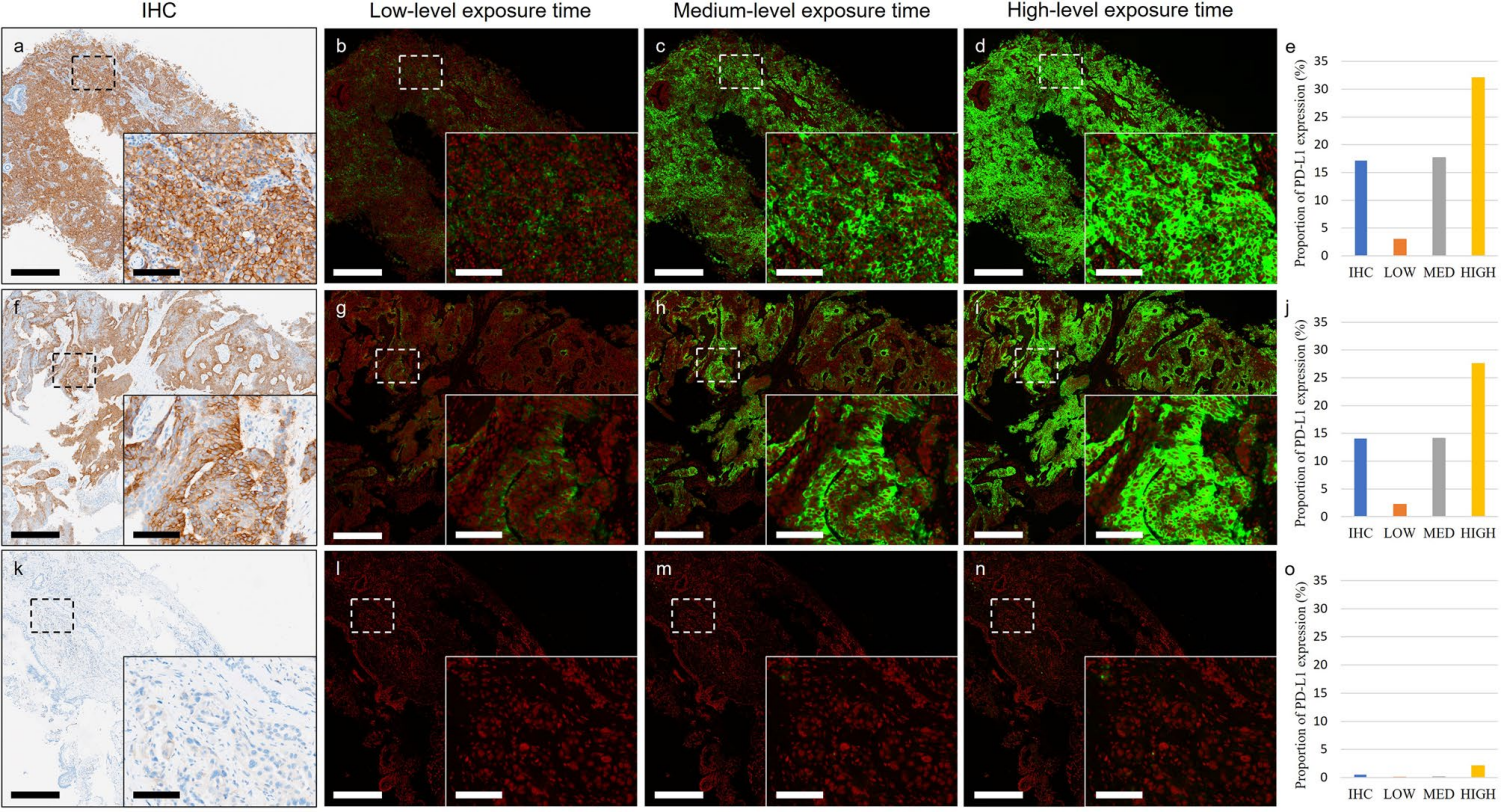
PD-L1 expression patterns in IHC and three IF images with different exposure time. Representative IHC images and three IF images of NSCLC slides with different exposure levels from three TPS categories with TPS of ≥ 50% (**a**), 1–49% (**f**), and < 1% (**k**). Zoom-in detailed view of dashed box area shows in lower right corner of images. (**b**,**g**,**l**) IF images captured with a low-level exposure time (6.5 ms). (**c**,**h**,**m**) IF images captured with a medium-level exposure time (25 ms). (**d**,**i**,**n**) IF images captured with a high-level exposure time (55 ms). (**e**) Proportion of PD-L1 expression areas from IHC and IF images of low/medium/high exposure time of specimens in (**a**–**d**). (**j**) Proportion of PD-L1 expression areas from IHC and IF images of low/medium/high exposure time of specimens in (**f**–**i**). (**o**) Proportion of PD-L1 expression areas from IHC and IF images of low/medium/high exposure time of specimens in (**k**–**n**). The proportion of PD-L1 expression areas of IHC and IF images were analyzed using computer-vision approach. *Green* PD-L1. *Red* nuclei. Scale bar: 500 μm; detailed view: 100 μm. (*LOW* low-level exposure time, *MED* medium-level exposure time; *HIGH* high-level exposure time.)

Adjustment of camera exposure time can ensure proper contrast and brightness during IF imaging^35^, crucial for clinical diagnosis to regulate imaging conditions and avoid artificial factors that may influence fluorescent signal levels. To establish a suitable exposure time for standard imaging process, we tested three levels: 6.5 ms (low-level), 25 ms (medium level), and 55 ms (high-level). The area proportion of PD-L1 expression in IF images with varying exposure time was calculated using a computer-vision approach, comparing their similarities with respective IHC images (Supplementary Fig. S3a). Median absolute errors to the IHC images were 9.32%, 1.16%, and 12.55% for IF images with low-, medium-, and high-level exposure times, respectively (Supplementary Fig. S3b). One-sided Student’s t-tests revealed that compared to IF images with low-level and high-level exposure times, those with medium-level exposure time demonstrated an area proportion of PD-L1 expression similar to IHC images (both *P* < 0.0003). IF images with medium-level exposure exhibited the most similar pattern to IHC across all three TPS categories, indicating consistency in visualization and computer‑vision analysis (Fig. 2). However, areas with faint PD-L1 expression in IHC remained challenging to detect. Parts with strong PD-L1 expression in IF images showed overexposure under medium-level exposure time (Fig. 2c,h), typically due to the limited dynamic range of the detection device^24^. Under limited hardware conditions, HDR processing was developed to address this limitation.

### Resolving dynamic range limitation by HDR processing for IF images

**Figure 3:**
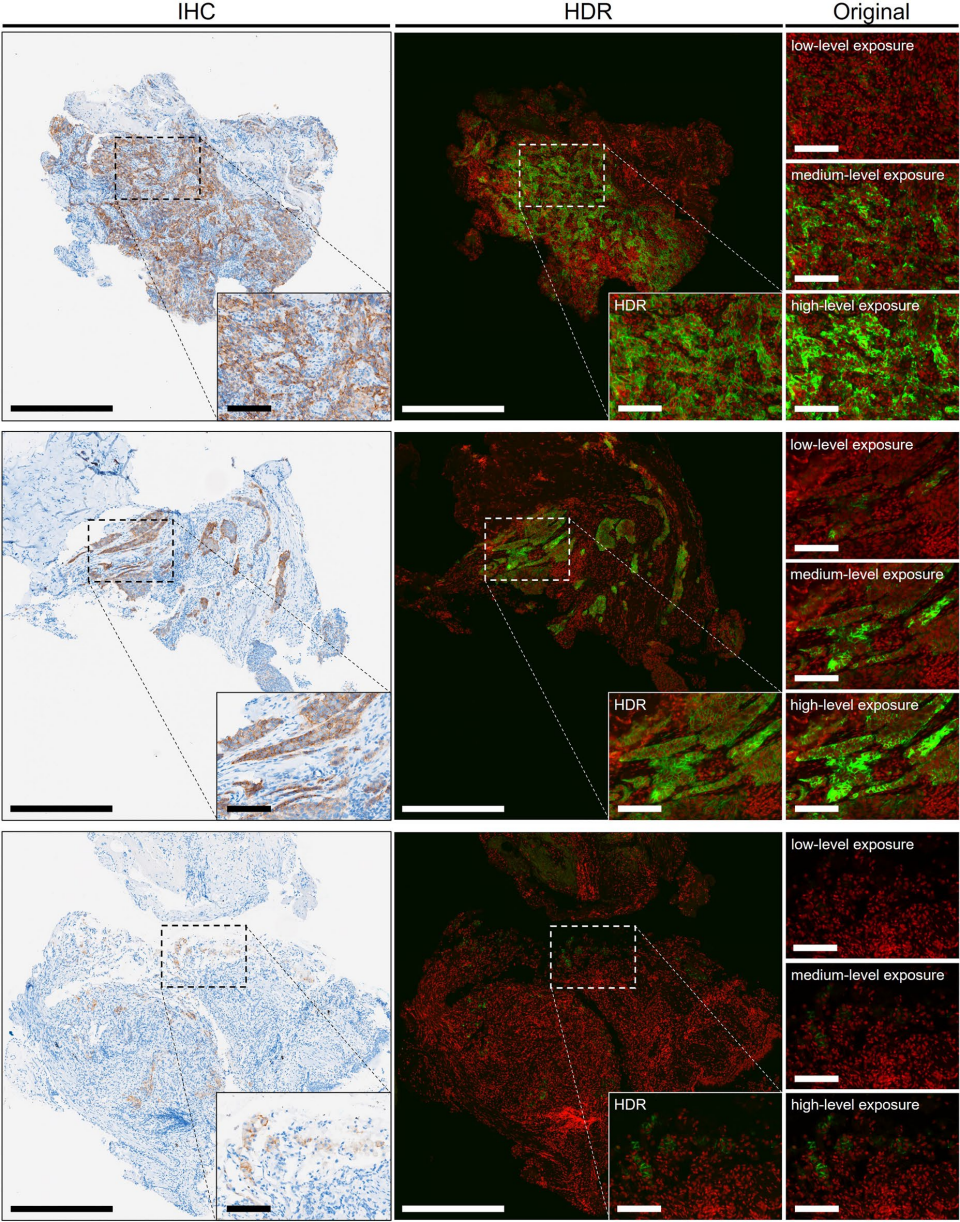
HDR images with similar pattern of PD-L1 expression to IHC. Representative HDR images (middle column) constructed from three original fluorescence images (right column) showing PD-L1 expression patterns similar to those of IHC images (left column) from the same patients. The original fluorescence images were obtained from three different exposure times: 6.5 (low-level), 25 (medium-level), and 55 ms (high-level). Zoom-in detailed view of dashed box area shows in lower right corner of images. These three cases were interpreted by pathologists in terms of TPS ≥ 50%, TPS 1–49%, and TPS < 1%, from top to bottom. *Green* PD-L1. *Red* nuclei. Scale bar: 500 μm; detailed view: 100 μm.

HDR processing was developed to integrate IF digital signals with varying intensities on the same slide under limited dynamic range of the detection device. To validate the HDR algorithm, images were analyzed with and without HDR processing using a computer‑vision approach, and similarity of the area proportion of PD-L1 expression was compared with that of IHC. HDR-processed images exhibited smaller overall error than those without HDR processing (Supplementary Fig. S4a,b), with median absolute errors of 0.15% and 1.16%, respectively. The data are shown in Supplementary Table S3. Significant differences were observed using one-sided Student’s t-test (*P* = 0.024), resolving the absence of weak and overexposed signals (Fig. 3). Comparing PD-L1 expression patterns between original florescence images and HDR-processed IF images, HDR images reflected the most similar PD-L1 pattern to IHC across all three TPS categories of representative specimens (Fig. 3).

For real-world PD-L1 diagnosis, we compared the variation in cases classified into the same TPS categories as IHC consensus. Pathologists’ scorings of IF images were weighed against the IHC consensus and measured using weighted kappa. After HDR processing, pathologist 2's weighted kappa value increased from 0.56 to 0.83, while pathologist 1’s remained stable (Table 1). With IF images showing significant accuracy improvement in TPS determination by pathologists, the weighted kappa values in the TPS 1–49% and ≥ 50% were raised from 0.71 to 0.79 and 0.29 to 0.86, respectively (Table 2).

**Table 1:** Classification of 28 NSCLC cases by individual pathologists.

		IHC consensus				IHC consensus		
	IF-MED	< 1%	1–49%	≥ 50%	HDR	< 1%	1–49%	≥ 50%
Pathologist 1	< 1%	11	1	0	< 1%	10	1	0
	1–49%	1	7	0	1–49%	2	8	0
	≥ 50%	0	3	5	≥ 50%	0	2	5
		Accuracy: 82.1% Weighted kappa: 0.78				Accuracy: 82.1% Weighted kappa: 0.78		
Pathologist 2	< 1%	12	7	0	< 1%	10	2	0
	1–49%	0	4	3	1–49%	2	9	0
	≥ 50%	0	0	2	≥ 50%	0	0	5
		Accuracy: 64.3% Weighted kappa: 0.56				Accuracy: 85.7% Weighted kappa: 0.83		

*IHC* immunohistochemistry, *IF* immunofluorescence, *MED* medium-level exposure time, *NSCLC* non-small cell lung cancer, *HDR* high dynamic range.

**Table 2:** Classification of 16 NSCLC cases with TPS ≥ 1% by individual pathologists.

Cases with TPS ≥ 1%		IHC consensus				IHC consensus		
	IF-MED	< 1%	1–49%	≥ 50%	HDR	< 1%	1–49%	≥ 50%
Pathologist 1	< 1%	0	1	0	< 1%	0	1	0
	1–49%	0	7	0	1–49%	0	8	0
	≥ 50%	0	3	5	≥ 50%	0	2	5
		Accuracy: 75% Weighted kappa: 0.71				Accuracy: 81.3% Weighted kappa: 0.79		
Pathologist 2	< 1%	0	7	0	< 1%	0	2	0
	1–49%	0	4	3	1–49%	0	9	0
	≥ 50%	0	0	2	≥ 50%	0	0	5
		Accuracy: 37.5% Weighted kappa: 0.29				Accuracy: 87.5% Weighted kappa: 0.86		

*IHC* immunohistochemistry, *IF* immunofluorescence, *MED* medium-level exposure time, *NSCLC* non-small cell lung cancer, *HDR* high dynamic range, *TPS* tumor proportion score.

Furthermore, interobserver agreement regarding TPS categories of IHC and IF images between pathologists was analyzed. The data are presented in Supplementary Table S4. Twenty-six of the 28 cases were classified into the same TPS categories (Table 3). IHC results demonstrated “almost perfect” agreement (weighted kappa = 0.91) between pathologists, indicating consistent diagnostic criteria for PD-L1 interpretation in IHC. Interobserver agreement regarding IF medium-level exposure images substantially improved after HDR processing, with 75% of cases classified into the same TPS categories by both pathologists, increasing weighted kappa from 0.39 to 0.69. As expected, HDR processing promoted interobserver agreement between pathologists in PD-L1 TPS diagnosis of IF images.

**Table 3:** Interobserver agreement between two pathologists in evaluating the TPS of IHC images, IF images with medium-level exposure time, and HDR images.

	IHC	IF-MED	HDR
All cases (n = 28)	26 (92.9%)	17 (60.7%)	21 (75%)
Weighted kappa	0.91	0.39	0.69
Interobserver reliability	Almost perfect	Fair	Substantial

*IHC* immunohistochemistry, *IF* immunofluorescence, *MED* medium-level exposure time, *HDR* high dynamic range, *TPS* tumor proportion score.

### Application of HDR method to 3D IF imaging

**Figure 4:**
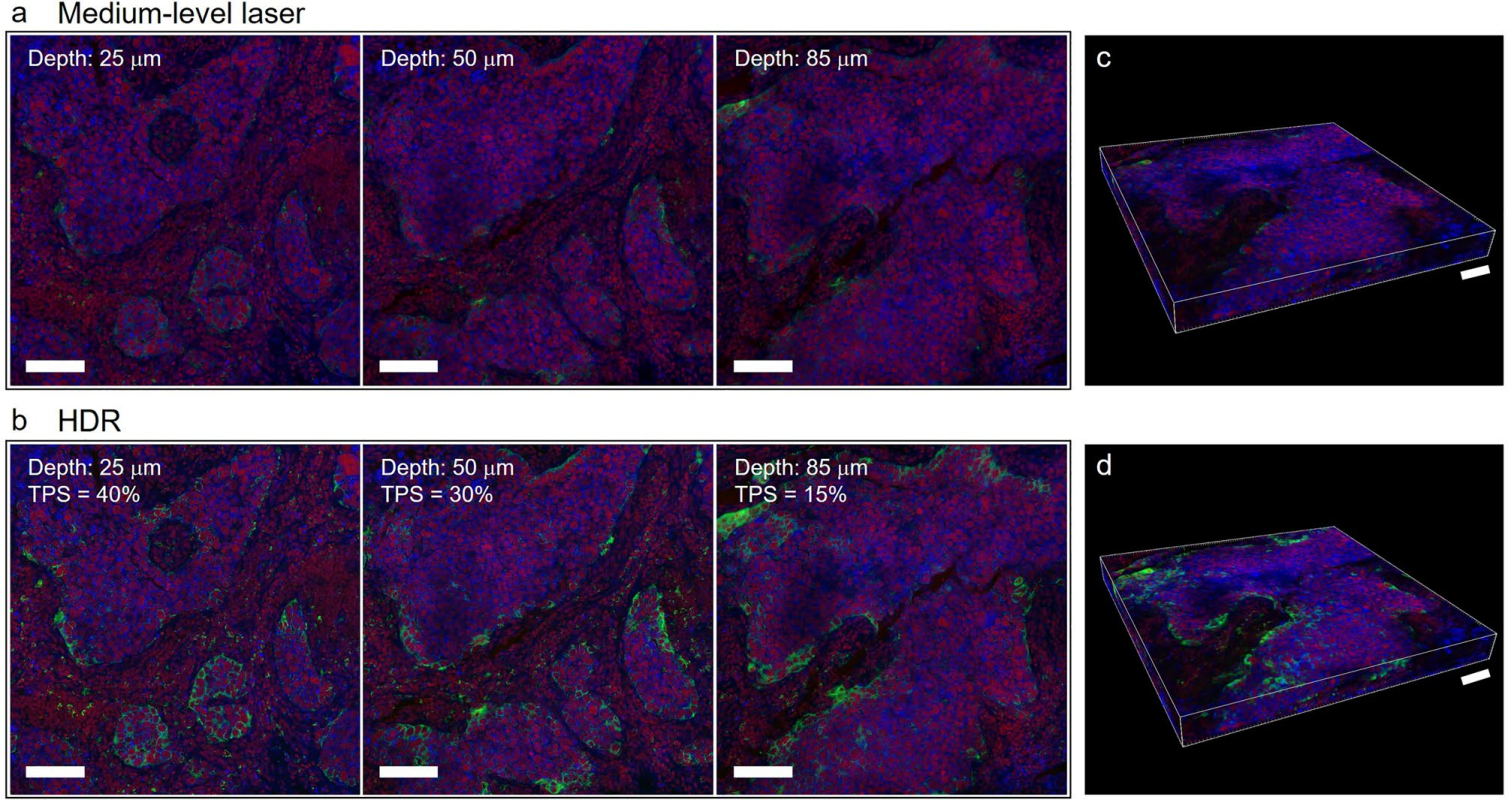
Applying HDR processing to 3D IF images for obtaining complete PD-L1 expression. (**a**) Images of NSCLC sample sections obtained using a medium-level laser at different depths: 25, 50, and 85 μm. (**b**) Complete PD-L1 expression is seen in HDR images at the superficial (depth: 25 μm), intermediate (depth: 50 μm), and bottom (depth: 85 μm) layers of the NSCLC samples. Maximal PD-L1 expression in tumor cells is seen at 25 μm depth of the tissue layer in the ROI image, and the 85 μm depth of tissue layer shows minimal PD-L1 expression, according to the pathologist’s scoring. (**c**) 3D rendering of NSCLC specimens using fluorescence images obtained using a medium-level laser. (**d**) 3D rendering of NSCLC specimens using fluorescence images obtained with HDR processing. *Green* PD-L1. *Red* nuclei. *Blue* plasma membrane. Scale bar: 100 μm; 3D rendering images: 100 μm.

We applied HDR processing to an 3D IF assay to depict comprehensive expression PD-L1 in 3D. Notably, we observed the loss of weak signals and overexposure of strong signals in 3D fluorescence imaging. However, with HDR processing, IF images exhibited complete shading in the PD-L1 pattern, displaying a distinct membrane staining pattern. Application of HDR processing across each 3D layer revealed actual PD-L1 expression and showed variability at different depths (Fig. 4a,b). Significant different PD-L1 expressions were observed at various depth levels in tumor tissue, with a TPS of 40% detected in the superficial layer and a TPS of 15% in the bottom layer. The HDR-processed serial images were reconstructed using 3D rendering (Fig. 4c,d). These results highlight the utility of HDR processing in both two-dimensional (2D) and 3D fluorescence imaging without necessitating hardware upgrades.

## Discussion

In this study, we demonstrate that the TSA system outperforms the fluorescent 2’Ab method in IF assays for pathological diagnosis. Adjusting exposure time during imaging also enhanced PD-L1 expression patterns in NSCLC specimens. However, the limited dynamic range of the detection device posed challenges in presenting complete PD-L1 patterns in IF images. Using HDR processing, we integrated PD-L1 signals captured with different exposure times into a single IF image to improve interobserver agreement of TPS evaluation between pathologists. Furthermore, 3D histological images of NSCLC tissue revealed the actual distribution of PD-L1 at various depths of the tumor environment. The modified staining, imaging, and post-processing of PD-L1 IF digital images were compatible with traditional IHC images, and could offer more precise visualization of PD-L1 expressions across different depths of tumor environments which could not be achieved by IHC tests.

Our IF digital images demonstrate that the TSA system closely mimics chromogen-based IHC patterns^36–38^, and is suitable for routine interpretation of IHC assays for immunotherapy without additional IF-specific guidelines. In the future, by considering the advantages of IF technique, such as multiplex staining on the same tissue slice for simultaneous observation of multiple biomarkers with their relative distribution using specific excitation and emission spectra of designed fluoroprobes^39^, pathologists will be able to study the tumor microenvironment more precisely, and provide more precise treatment suggestions for patients with non-small lung cancer.

On the other hands, the limitation of microscope detection devices was reduced fluorescent signal visibility, leading to incomplete interpretation in digital images. Advanced detectors can expand the dynamic range of signal detection, but their high cost limits their use in routine clinical practices^40,41^. HDR processing has been applied in various studies to overcome imaging limitations, including embryonic anatomy and organ function evaluation^23,42–44^. We applied a modified HDR algorithm to improve the assessment of IF expression specialized for pathological interpretation. The proposed HDR algorithm differs from existing approaches is that it includes both pre-processing and post-processing procedures to enhance the contrast of membrane staining. Furthermore, this algorithm for constructing fluorescent HDR images remains lightweight and accessible to general laboratories with basic microscopes to improve the clarity of morphological patterns for the expression of PD-L1. Regarding interobserver agreement between pathologists for TPS (Table 3), we found that the agreement of TPS in IF images after HDR processing was ameliorated, but still had not as much as the agreement in IHC, possibly because IF images were less frequently used in routine training of pathologists. Except for additional training and more clinical cases, we can also apply pseudo-H&E and IHC processing to transform the color domain of fluorescent images, which will help pathologists become more familiar with fluorescent images to evaluate the biomarker expression patterns in a familiar format^45^.

In clinical practice, PD-L1 scoring may result in inaccurate stratification for immunotherapy because of tumor heterogeneity and limited morphological information from 2D IHC^46,47^. Optical clearing techniques and advanced confocal microscopy enable 3D pathology studies more feasible, and avoid the limitations from insufficient tumor cells in 2D tissue sections. Our 3D PD-L1 analysis provides at least 25 times more pathological information than conventional H&E/IHC by using the 100-μm-thick sections. This advantage can further facilitate more accurate correlation between PD-L1 expression and immunotherapy response. In a previous study, these techniques were compatible with a computer-assisted algorithm and revealed the variability in PD-L1 distribution in 3D NSCLC tissues^25^. We integrated 3D IF assay with HDR processing to quantify actual PD-L1 expression and distribution in 3D NSCLC specimens at different depths. Despite limited sample size, we found 25% of PD-L1 tumor expression level was changed in the tumor tissue. Moreover, computer-assisted prediction algorithms could be integrated into future clinical pathologic practice to assist in the accurate identification and quantitation of tumor PD-L1 expression^48^.

In conclusion, the versatility of the TSA system with adjusted imaging conditions and HDR processing was applicable in both 2D and 3D pathologic evaluation of PD-L1 expression in tumor tissue of NSCLC with good clinical quality. The complete preparation process of digital IF images proved its potential for routine evaluation of NSCLC with different levels of PD-L1 expression in different depths of lung tumor tissue. Our work provides a systematic method to obtain higher accuracy for PD-L1 expression assessment in non-small cell lung cancer under limited dynamic range of the detection device. It would be a feasible tool to promote the utilization of fluorescent microscopy in general laboratory or medical center. Our work supports more accurate 3D pathological analysis of PD-L1 distribution in NSCLC tumor tissues and provides an alternative solution for the study of heterogeneous distribution of PD-L1. The method reported in this paper can be extended to other clinical biomarker observation for improving the sensitivity. Finally, our new methodology will promote precision medicine in diagnostic strategies for lung cancer and shed a light for more precise choice in patients with lung cancer for immunotherapy therapy in the near future.

### Supplementary Information

Supplementary Information.

## Data Availability

The datasets generated during and/or analyzed during the current study are available from the corresponding author on reasonable request. The implementation of our HDR algorithm can be found at https://github.com/JelloXBiotechInc/immunofluorescenceHDR.
