# Supplementary material for: Optimizing immunofluorescence with high-dynamic-range imaging to enhance PD-L1 expression evaluation for 3D pathology assessment from NSCLC tumor tissue
Source: Sci Rep. 2024 Jul 2;14:15176. doi: 10.1038/s41598-024-65187-x (PMC11219731; doi:10.1038/s41598-024-65187-x)
Supplement: Supplementary file 1 — Supplementary Information. [file 41598_2024_65187_MOESM1_ESM.pdf]

Supplementary Information

Optimizing Immunofluorescence with High-Dynamic-Range Imaging to Enhance PD-L1 Expression Evaluation for 3D Pathology Assessment from NSCLC Tumor Tissue

Hsien-Neng Huang[1, 2], Chun-Wei Kuo[1], Yu-Ling Hung[3], Chia-Hung Yang[3], Yu-Han Hsieh[3], Yu-Chieh Lin[3], Margaret Dah-Tsyr Chang[3], Yen-Yin Lin[3], Jen-Chung Ko[4*]

[1] *Department of Pathology, National Taiwan University Hospital Hsin-Chu Branch,* Hsinchu, Taiwan

[2] Department and Graduate Institute of Pathology, College of Medicine, National Taiwan University, Taipei, Taiwan

[3] JelloX Biotech Inc., Hsinchu, Taiwan

[4] *Department of Internal Medicine, National Taiwan University Hospital Hsin-Chu Branch,* Hsinchu, Taiwan

Supplementary Materials

Detailed description of the modified version of high dynamic range algorithm used in this study

Here, a detailed step-by-step description of the modified high dynamic range (HDR) algorithm proposed in the current study is presented.

1. Given the immunofluorescence (IF) images of a specimen collected with different exposures, SYTO and programmed death ligand 1 (PD-L1) channels were separated. Note that the different exposures only affected the PD-L1 channels, and their SYTO channels remained identical.
2. A collection of "pseudo images" was generated by preprocessing the PD-L1 channels of the original IF images by erosion and Gaussian blurring of kernel sizes 5 and 3, respectively. These pseudo images had the same SYTO channels and exposures as the corresponding original images.
3. A binary mask of the tissue area was obtained via the subsequent dilation of a kernel size of 21 and Otsu thresholding on the SYTO channel.
4. Let m be the total number of original and pseudo images. N pixel locations were sampled from the tissue area, such that n is the smallest integer greater than 255 / (m – 1), and all these pixels had mean values greater than 16 in the original and pseudo images.
5. A response curve was fitted by minimizing the quadratic objective suggested in the report by Debevac and Malik with singular value decomposition and the sampled pixel values in the original and pseudo images, except that the weighting function $w(z)$ was offset by 16, and the regularization parameter λ was set to 100.
6. The irradiance of the PD-L1 channel of the underlying specimen was estimated using equation 6 in the report by Debevac and Malik,[1] with the offset weighting function and the fitted response curve obtained in step 5.

7. The "merged" PD-L1 channel was determined by linearly scaling the irradiance estimated in step 6 between the minimum and maximum pixel values of the PD-L1 channels of the original and pseudo images.
8. A threshold α was determined by the 99.9th-quantile of the merged PD-L1 channel in the non-tissue area (which had been computed in step 3), and pixels with merged PD-L1 values x greater than α were offset by $(255 - \alpha)[(x - \alpha)/(255 - \alpha)]^{1/\gamma}$, where $\gamma = 1.3$.
9. The merged PD-L1 channel was post-processed using contrast-limited adaptive histogram equalization on an 8-by-8 grid,[2] and the maximum histogram density was limited to 3.
10. The merged PD-L1 channel was post-processed via subtraction by its black-hat transformation and addition by white-hat transformation, using an ellipse kernel of size 3. This contrast enhancement procedure was performed twice, and between consecutive enhancements, Gaussian blurring of a kernel size of 3 was applied.
11. Finally, the merged PD-L1 channel was combined with the input SYTO channel to produce an HDR image of the specimen.

PD-L1 expression threshold computation in the computer-vision analysis

PD-L1 expression histograms of the IF slides, immunohistochemistry (IHC) slide, and HDR images were collected, where the former histogram was the aggregation over those of slides with different exposures. The Li threshold was first computed from the histogram of IF slides.[3] Next, this Li threshold was projected onto the histograms of the IHC slide and HDR image through subsequent histogram equalization and its inverse procedure. In other words, these thresholds shared a similar percentile in their

respective histograms. Additionally, the threshold values were limited to a minimum during threshold computation from and projection onto histograms to avoid background noise. This minimum threshold was set to 8 and 64 for the histograms of the IF images and the IHC slide, respectively.

References

1. Debevec PE, Malik J. Recovering high dynamic range radiance maps from photographs. In: *ACM SIGGRAPH 2008 classes*. 2008:1–10.
2. Pizer SM, et al. Adaptive histogram equalization and its variations. *Comput Vis Graph Image Process*. 1987;39:355–368. doi:10.1016/S0734-189X(87)80186-X.
3. Li CH, Lee CK. Minimum cross entropy thresholding. *Pattern Recogn.* 1993;26:617–625. doi:10.1016/0031-3203(93)90115-D.

Supplementary Tables

Table S1. Conditions for three-dimensional histopathology imaging acquisition using an FV3000 confocal laser scanning microscope.

**Condition of SYTO16 channel**			
Sensitivity (HV)	390		
Offset	3		
Depth of tissue section (μm)	10	53	120
Laser intensity (%)	0.03	0.08	0.26
**Condition of DiD channel**			
Sensitivity (HV)	390		
Offset	3		
Depth of tissue section (μm)	10	53	120
Laser intensity (%)	0.7	1.3	3.1
**Condition of Alexa Fluor 555 channel**			
Sensitivity (HV)	390		
Offset	3		
Depth of tissue section (μm)	10	53	120
Laser intensity (%)			
Weak level	0.03	0.37	0.49
Medium level	0.08	0.42	0.54
Strong level	0.19	0.53	0.65

* Laser intensity changed with a fixed slope with depth variation of tissue sections.

Table S2. PD-L1 TPS of NSCLC specimens evaluated by two pathologists using IHC and IF images with medium-level exposure time as well as HDR processing.

	**IHC TPS**			**IF-MED TPS**		**IF-HDR TPS**	
**Case ID.**	Pathologist 1	Pathologist 2	Consensus	Pathologist 1	Pathologist 2	Pathologist 1	Pathologist 2
1	TPS <1%	TPS <1%	TPS <1%	TPS <1%	TPS <1%	TPS <1%	TPS <1%
2	TPS ≥50%	TPS ≥50%	TPS ≥50%	TPS ≥50%	TPS ≥50%	TPS ≥50%	TPS ≥50%
3	TPS 1-49%	TPS 1-49%	TPS 1-49%	TPS 1-49%	TPS 1-49%	TPS 1-49%	TPS 1-49%
4	TPS ≥50%	TPS ≥50%	TPS ≥50%	TPS ≥50%	TPS ≥50%	TPS ≥50%	TPS ≥50%
5	TPS 1-49%	TPS 1-49%	TPS 1-49%	TPS 1-49%	TPS 1-49%	TPS ≥50%	TPS1-49%
6	TPS <1%	TPS <1%	TPS <1%	TPS <1%	TPS <1%	TPS <1%	TPS <1%
7	TPS 1-49%	TPS 1-49%	TPS 1-49%	TPS 1-49%	TPS <1%	TPS 1-49%	TPS 1-49%
8	TPS 1-49%	TPS 1-49%	TPS 1-49%	TPS 1-49%	TPS <1%	TPS 1-49%	TPS 1-49%
9	TPS 1-49%	TPS 1-49%	TPS 1-49%	TPS ≥50%	TPS 1-49%	TPS ≥50%	TPS 1-49%
10	TPS 1-49%	TPS <1%	TPS 1-49%	TPS 1-49%	TPS <1%	TPS 1-49%	TPS 1-49%
11	TPS 1-49%	TPS 1-49%	TPS 1-49%	TPS 1-49%	TPS <1%	TPS 1-49%	TPS <1%
12	TPS <1%	TPS <1%	TPS <1%	TPS 1-49%	TPS <1%	TPS <1%	TPS <1%
13	TPS <1%	TPS <1%	TPS <1%	TPS <1%	TPS <1%	TPS 1-49%	TPS 1-49%
14	TPS 1-49%	TPS 1-49%	TPS 1-49%	TPS 1-49%	TPS 1-49%	TPS <1%	TPS 1-49%
15	TPS <1%	TPS <1%	TPS <1%	TPS <1%	TPS <1%	TPS <1%	TPS <1%
16	TPS <1%	TPS <1%	TPS <1%	TPS <1%	TPS <1%	TPS <1%	TPS <1%
17	TPS <1%	TPS <1%	TPS <1%	TPS <1%	TPS <1%	TPS <1%	TPS <1%
18	TPS ≥50%	TPS ≥50%	TPS ≥50%	TPS ≥50%	TPS 1-49%	TPS ≥50%	TPS ≥50%

19	TPS <1%	TPS <1%	TPS <1%	TPS <1%	TPS <1%	TPS <1%	TPS 1-49%
20	TPS <1%	TPS <1%	TPS <1%	TPS <1%	TPS <1%	TPS <1%	TPS <1%
21	TPS ≥50%	TPS 1-49%	TPS ≥50%	TPS ≥50%	TPS 1-49%	TPS ≥50%	TPS ≥50%
22	TPS 1-49%	TPS 1-49%	TPS 1-49%	TPS ≥50%	TPS <1%	TPS 1-49%	TPS <1%
23	TPS 1-49%	TPS 1-49%	TPS 1-49%	TPS ≥50%	TPS <1%	TPS 1-49%	TPS 1-49%
24	TPS <1%	TPS <1%	TPS <1%	TPS <1%	TPS <1%	TPS <1%	TPS <1%
25	TPS ≥50%	TPS ≥50%	TPS ≥50%	TPS ≥50%	TPS 1-49%	TPS ≥50%	TPS ≥50%
26	TPS <1%	TPS <1%	TPS <1%	TPS <1%	TPS <1%	TPS <1%	TPS <1%
27	TPS 1-49%	TPS 1-49%	TPS 1-49%	TPS <1%	TPS <1%	TPS 1-49%	TPS 1-49%
28	TPS <1%	TPS <1%	TPS <1%	TPS <1%	TPS <1%	TPS 1-49%	TPS <1%

Note: Cases 11 and 23 were excluded because the number of tumor cells in sections was < 100.

PD-L1, programmed death ligand 1; TPS, tumor proportion score; NSCLC, non-small cell lung cancer; IHC, immunohistochemistry; IF, immunofluorescence; MED, medium-level exposure time; HDR, high dynamic range.

Table S3. Full data of annotated tumor area (μm^2) and PD-L1 expressed area (μm^2) of IHC images, three original IF images, and HDR-processed IF images for 15 specimens.

	Annotated tumor area		PD-L1 expression area				
**Case ID**	IHC	All IF	IHC	IF-LOW	IF-MED	IF-HIGH	IF-HDR
2	1868443.04	1910434.49	460464.33	91705.78	492915.12	851451.54	475400.21
4	66362302.56	67004248.73	11351562.33	1980945.23	11816293.24	21469680.95	11433437.35
6	2038913.53	2130123.74	10188.95	50.41	2645.16	44413.63	10468.95
7	2009019.56	2093915.17	79603.23	2207.79	58419.88	250975.98	84568.59
8	1645067.04	1601656.70	169105.46	7558.83	127522.07	454814.12	168120.31
9	38586111.50	38708341.77	5420113.81	853986.77	5451304.64	10670429.07	5416469.24
10	3476734.97	3622875.34	483849.49	13776.91	283569.15	1650039.08	528381.96
13	707321.71	707986.66	23753.61	77.27	15687.92	76438.78	24850.56
17	199127.87	201061.03	1352.27	0.00	334.89	6498.54	1396.48
18	602664.91	588712.61	7295.00	0.00	217.50	43602.56	7964.13
19	901176.72	842050.69	151642.27	10745.38	129319.83	303664.73	143960.36
22	1137085.11	1056250.81	122680.66	15454.56	104588.67	246489.75	116165.48
25	1400417.32	1412953.61	91414.96	13710.76	72910.36	220775.93	97025.82
27	26925227.63	28520457.80	4195606.12	565650.70	4483332.37	8847449.62	4545478.77
30	817534.62	875990.05	21426.17	603.04	9257.66	96585.13	22128.83

PD-L1, programmed death ligand 1; IHC, immunohistochemistry; IF, immunofluorescence; HDR, high dynamic range; LOW, low-level exposure time; MED, medium-level exposure time; HIGH, high-level exposure time.

Table S4. Interobserver agreement between pathologists' evaluation of TPS by viewing 28 cases of IHC, IF images with medium-level exposure time, and HDR images.

A. Comparison of IHC TPS between pathologists.

	Pathologist 2		
Pathologist 1	<1%	1- 49%	≥50%
<1%	12	0	0
1- 49%	1	10	0
≥50%	0	1	4

B. Comparison of IF-MED TPS between pathologists.

	Pathologist 2		
Pathologist 1	<1%	1- 49%	≥50%
<1%	12	0	0
1- 49%	5	3	0
≥50%	2	4	2

C. Comparison of HDR TPS between pathologists.

	Pathologist 2		
Pathologist 1	<1%	1- 49%	≥50%
<1%	9	2	0
1- 49%	3	7	0
≥50%	0	2	5

IHC, immunohistochemistry; IF, immunofluorescence; HDR, high dynamic range; MED, medium-level exposure time; TPS, tumor proportion score.

Supplementary Figures

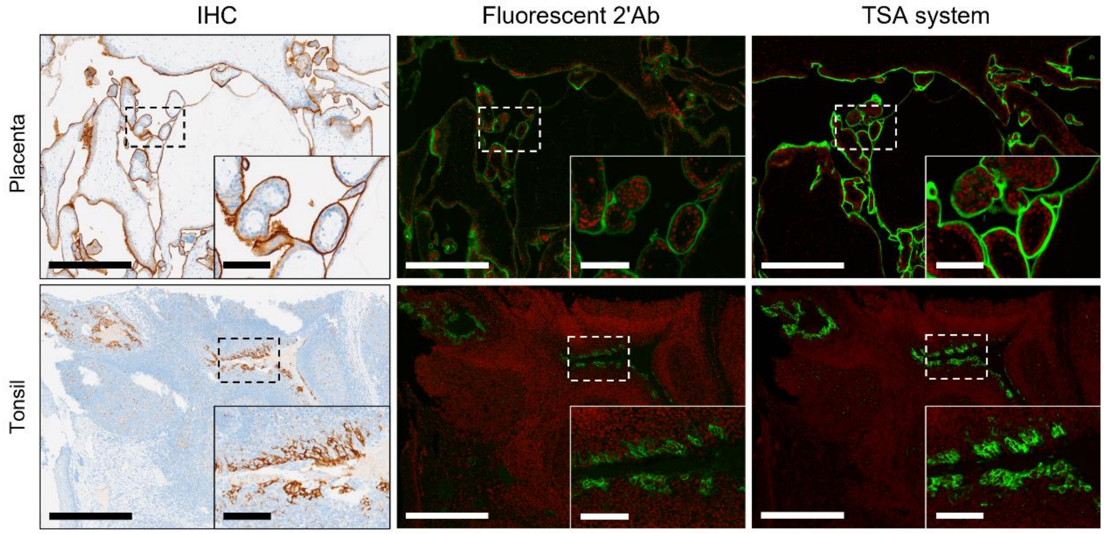

Fig S1. Human placental and tonsil tissues used as positive controls for PD-L1 staining. Membranous expression patterns of PD-L1 in human placenta and tonsil tissues showing similarity IHC patterns in both IF images using the fluorescent 2′Ab and TSA methods. *Green*: PD-L1. *Red*: nuclei. Scale bar: 500 μm; detailed view: 100 μm.

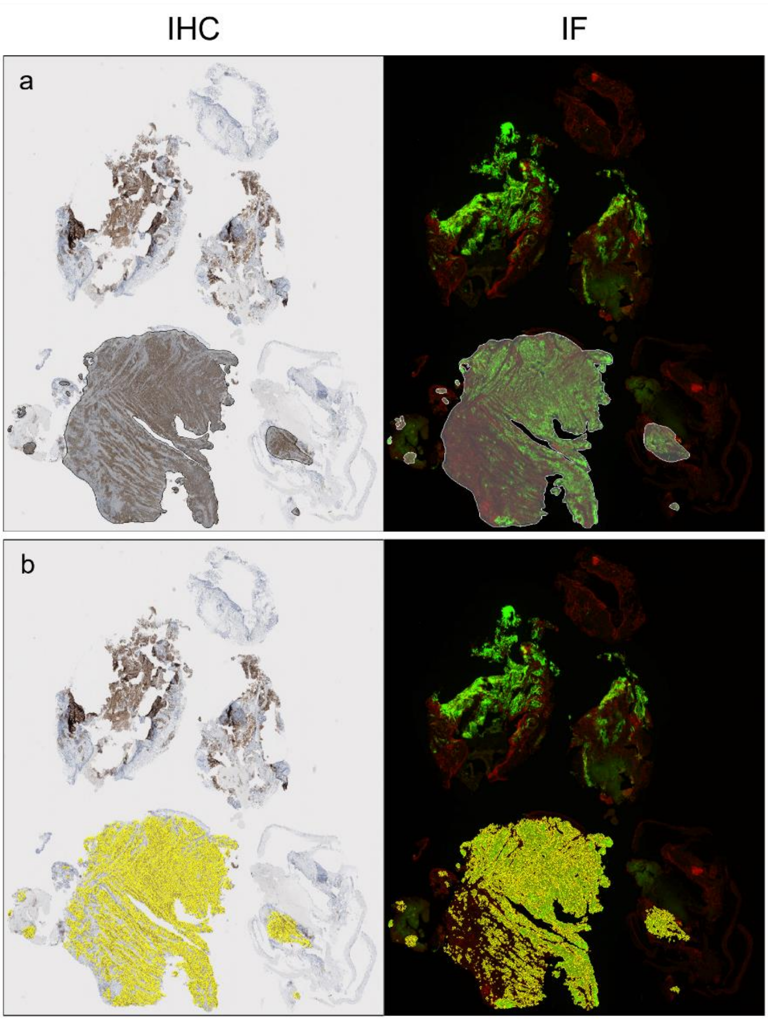

Fig S2. Manual annotation and PD-L1 expression analysis of the IHC and IF images. (a) Corresponding areas in both IHC and IF images that contained most of the tumor were annotated (white mask), where ruptured and folded regions were avoided. (b) PD-L1 expression within the annotated regions (yellow mask) was determined using a computer-vision approach. *Green*: PD-L1. *Red*: nuclei.

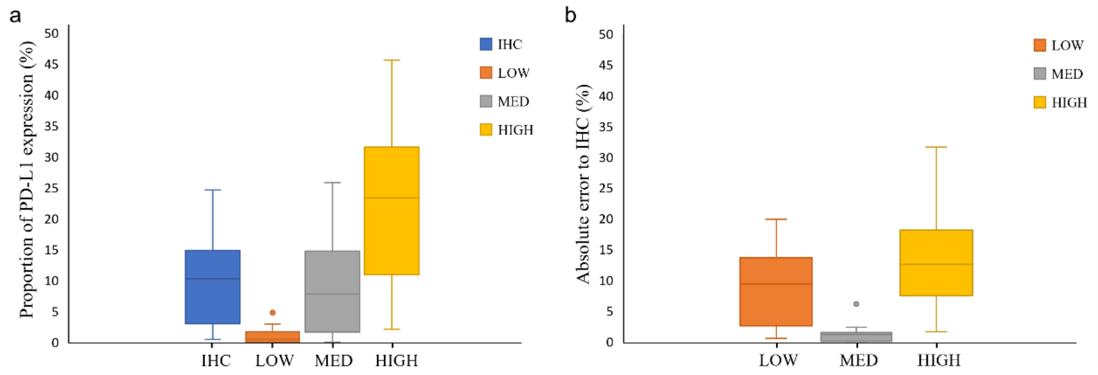

Fig S3. Comparison of PD-L1 expression using computer-vision analysis in IHC and three IF images with different exposure times. (a) Proportion of PD-L1 expression areas of the IHC images and the IF images with a low/medium/high-level exposure time is calculated using the computer-vision approach. For each group of images, fifteen NSCLC samples are acquired. (b) The absolute value of the difference in expression area proportion to the IHC images is calculated. The whiskers of the boxplot show the minimum and maximum values for each group. (LOW, low-level exposure time; MED, medium-level exposure time; HIGH, high-level exposure time.)

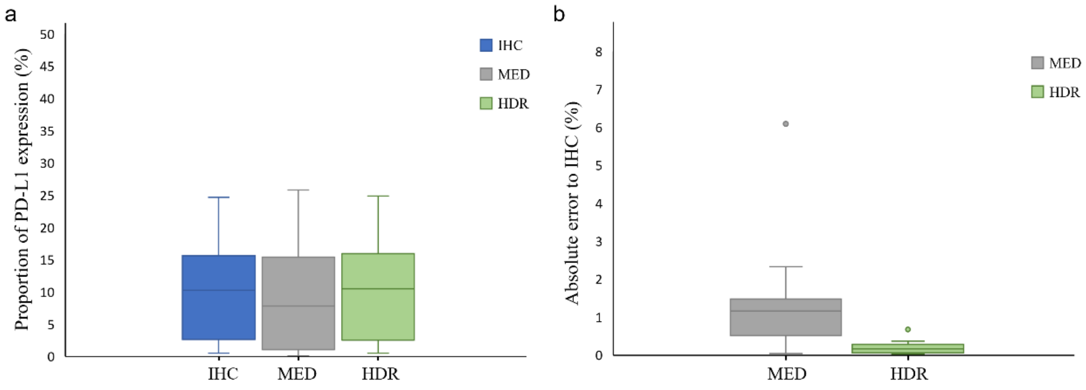

Fig S4. Computer-vision analysis of PD-L1 expression in IHC, IF images with medium-level exposure, and HDR images of NCSLC specimens. (a) Proportion of PD-L1 expression areas of 15 NSCLC samples from the IHC images, and IF images with medium-level exposure time, and HDR images is calculated using the computer-vision approach. (b) The absolute value of expression area proportion difference to the IHC images is calculated. (MED, medium-level exposure time; HDR, high dynamic range.)
